# *Pseudomonas aeruginosa* necrotizing bronchopneumonia

**DOI:** 10.4322/acr.2021.271

**Published:** 2021-05-06

**Authors:** Ranjit I Kylat

**Affiliations:** 1 University of Arizona, College of Medicine, Department of Pediatrics, Tucson, Arizona, USA

**Keywords:** Pseudomonas aeruginosa, Bronchopneumonia, Infant, Premature, Infant, Low Birth Weight, Neonatal Sepsis, Cross Infection

## Abstract

Extremely low birth weight (ELBW) infants are at particularly high risk for infection due to an immature immune system, invasive procedures such as endotracheal intubation, intravascular catheterization, and other factors. Neonatal infections in this population are associated with a high mortality, poor growth, and neurodevelopmental outcomes. *Pseudomonas aeruginosa* (*P. aeruginosa*) infection is an uncommon but potentially devastating cause of pneumonia and sepsis in the ELBW population. *P. aeruginosa* is an important cause of healthcare-associated infections (HAI) or nosocomial infections. *P. aeruginosa* can perceive unfavorable environmental changes and orchestrate adaptations by developing plasmid-mediated and adaptive resistance to antibiotics. We describe an ELBW infant born at 26 weeks’ gestation who succumbed at 13 days of life to *P. aeruginosa* infection. Some of the factors related to the pathogenesis and multidrug resistance are described.

## INTRODUCTION

Sepsis is a systemic inflammatory response syndrome in the presence of infection.^1^ Neonatal sepsis is classified as early or late-onset, based on the onset of its occurrence, before or after 72 hours of life. In the U.S., bacterial sepsis is a leading cause of neonatal mortality, affecting 32,000 live births annually.^1^ ELBW infants are at particularly high risk for infection due to immature immune system, prolonged endotracheal intubation, presence of intravascular catheters, and other factors.^2^^,^^3^ Neonatal infections in this population are associated with a high mortality, poor growth, and neurodevelopmental outcomes.^1^^-^^3^ *P. aeruginosa* infection is an uncommon but potentially devastating cause of pneumonia and sepsis in the extreme preterm population. This gram-negative, encapsulated, aerobic, rod-shaped bacterium belongs to the family of Pseudomonadaceae, which is in the class of gammaproteobacteria.^4^^,^^5^ It is recognized for being an opportunist pathogen, usually infecting an immunocompromised host.^4^^,^^5^ Although, generally, it is aerobic, it is also a facultative anaerobe, as it is well adapted to proliferate in conditions of partial or total oxygen depletion.^4^^,^^5^ *P. aeruginosa* is an important cause of healthcare-associated infections (HAI).^6^ An estimated 5-15% of all hospitalized patients experience an HAI, and the emergence of multi-drug resistance (MDR) has added to the devastating effects of *P. aeruginosa*. The U.S. Centers for Disease Control estimates that approximately every year 2 million infections and 90,000 deaths are contracted in U.S. healthcare facilities, resulting in an estimated $4.5 billion in excess medical costs.^7^ In a study of 41.6 million hospitalized patients in a cohort of 890 U.S. hospitals, the incidence for MDR *P. aeruginosa* infection has only marginally decreased from 13.1 to 9.4 per 10,000 hospitalizations from 2012 to 2017.^8^ Herein, we describe a patient with late-onset sepsis due to *P. aeruginosa*.

## CASE REPORT

A 32-year-old primigravida was hospitalized for premature rupture of membranes and variable fetal decelerations at 25 weeks’ gestation. She received prenatal steroids and antibiotics with ampicillin and azithromycin for premature, prolonged rupture of membranes. All her prenatal screening tests were normal, except for group B streptococcal screening, which was not available. However, she developed hypertension, elevated liver enzymes, and thrombocytopenia. An ultrasound examination at that time revealed the baby to be in a transverse position, and a cesarean section was performed. A female infant was delivered at 26 weeks gestational age with a birth weight of 630 g. She showed some signs of spontaneous respiration but was unable to oxygenate, requiring orotracheal intubation, mechanical ventilation, and surfactant administration. Her APGAR scores were 6 and 7 at 1 and 5 minutes, respectively. Her hospital course was complicated by a hemodynamically significant patent ductus arteriosus (PDA), for which she treated with ibuprofen. However, although it reduced in size, the patency remained small to moderate with a left to right shunt. Apart from her PDA, the infant remained stable until the thirteenth day, when she experienced respiratory decompensation and decreasing hematocrit. After obtaining blood culture, due to the clinical suspicion of sepsis, she was started on broad-spectrum antibiotics, cefotaxime, and vancomycin. Her small volume of enteral feeding was discontinued, and she was given only parenteral nutrition. Blood-stained aspirates from her endotracheal tube raised the concern for pulmonary hemorrhage. There was also evidence of thrombocytopenia and coagulopathy for which she received multiple blood products, including platelets, red cells, and frozen plasma. Over eight hours, she continued to require increased ventilatory settings, and the antibiotic, cefotaxime was changed to meropenem. The patient developed hypotension and needed multiple vasopressors, including dopamine, epinephrine, and subsequently corticosteroids, as the hypotension remained refractory. The patient continued to get progressively worse for the next twelve hours with profound metabolic acidosis and anuria. After discussions with the family, a decision to withdraw critical care support was made. The clinical diagnosis at that point was extreme prematurity and severe sepsis with multi-organ failure. The blood culture drawn prior to initiation of antibiotics grew *P. aeruginosa,* and the result obtained 16 hours after the patient’s demise revealed susceptibility only to imipenem and cefepime. The family consented to an autopsy.

## AUTOPSY

At autopsy, the infant appeared premature at around 26 weeks gestational age with periorbital and dependent edema. The body weighed 785 gm with 1 to 2 cc serous fluid in pleural cavities and 15 cc of serous fluid within the peritoneal cavity.

The cardiovascular examination revealed a small ductus arteriosus. The great vessels and heart chambers were in a normal anatomic relationship. Microscopic examination of the heart showed immature myocytes.

**Figure 1:**
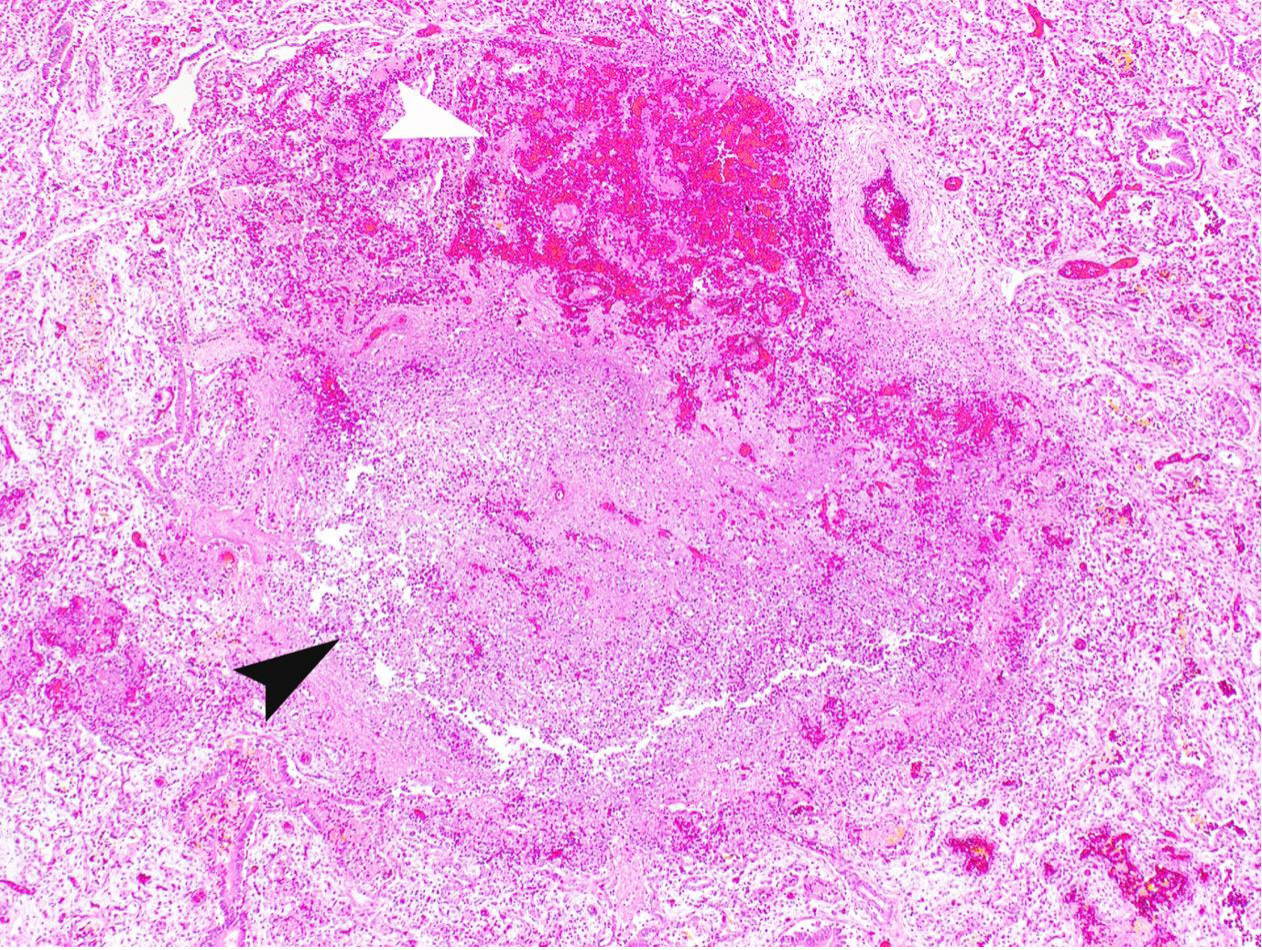
Photomicrograph of the lung. Lung parenchyma with areas of severe hemorrhage (thin white arrowhead) and necrotizing abscess (black arrowhead) (H&E; 4X).

The pleura showed multiple small foci of hemorrhage and petechiae. The lung parenchyma was dark red-brown with multifocal scattered hemorrhages measuring up to 0.4 cm. On histopathological examination, the lung tissue was in the canalicular stage of development. Multiple foci of necrotizing abscesses, fibrin deposition, hyaline membrane, acute inflammation, and hemorrhage were present throughout the parenchyma with yellow granular material collections within the alveoli. (Figures 1, 22B).

**Figure 2:**
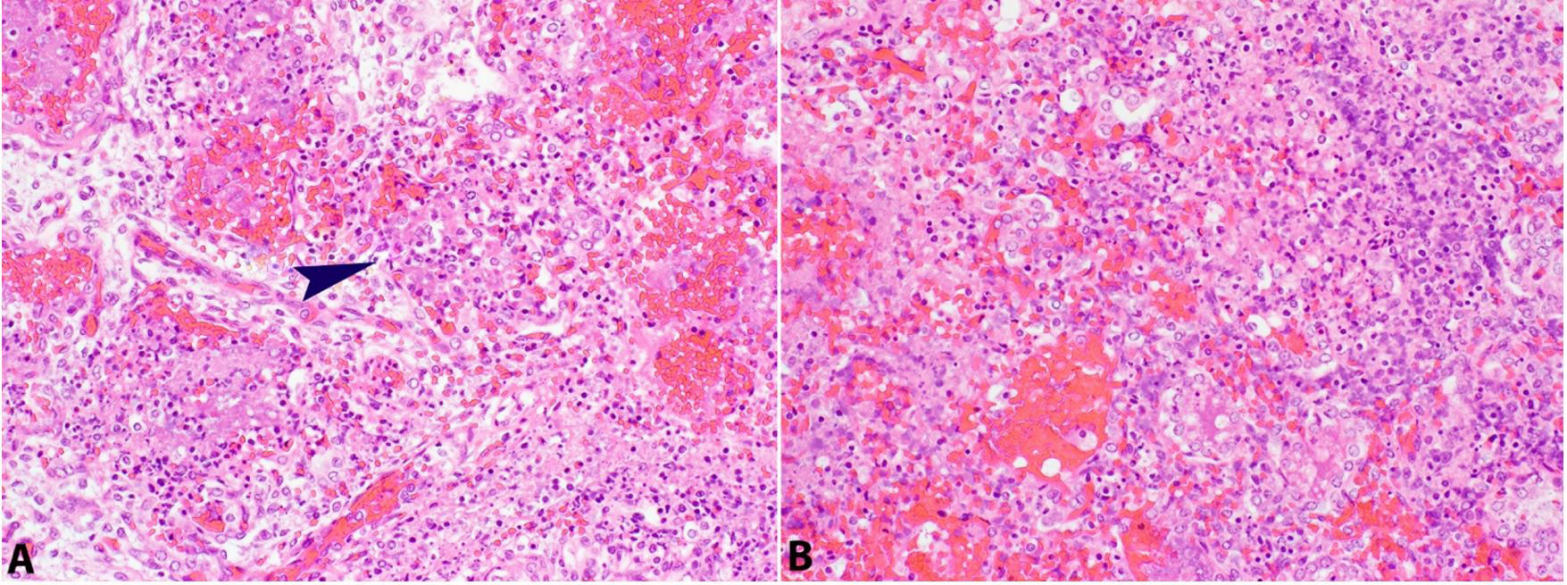
Photomicrographs of the lung. **A –** Right lung parenchyma with extensive acute inflammation and fibrin deposition (arrow) (H&E; 20X); **B –** Left lung parenchyma with hemorrhage, acute inflammation and fibrin deposition (H&E; 20X).

The digestive system examination was unremarkable. The liver surface was brown-red with some dark red to purple-gray mottling. On section, the liver parenchyma was reddish-brown and showed extra-medullary hematopoiesis and sinusoidal congestion. The complete examination of the musculoskeletal, genitourinary, hematopoietic, nervous, and endocrine systems was normal. Microbiologic cultures of the blood from the heart chamber and lung grew *P. aeruginosa*.

The final pathologic diagnosis was bilateral acute necrotizing bronchopneumonia with abscess formation and likely due to septicemia due to *P. aeruginosa*. The underlying cause of death was extreme prematurity and the need for prolonged mechanical ventilation.

## DISCUSSION

Late-onset neonatal sepsis is acquired from the environment, which includes the gut microbiome.^9^^,^^10^ Apart from extreme prematurity, the other risk factors include prolonged hospitalization, indwelling vascular catheters, medications such as proton pump inhibitors and histamine-2 blockers.^9^^,^^10^ Clinical signs are extremely variable and include a range of multisystem effects such as temperature instability, lethargy, irritability, apnea, acidosis, intolerance to feed, and cardio-respiratory decompensation. Leukocytosis, leukopenia, neutropenia, high immature or band cell counts, and abnormalities in acute phase reactants may be seen, but the isolation of the microorganism from a sterile body site (such as blood or cerebral spinal fluid) is the gold standard for diagnosis.^2^^,^^9^^,^^10^

*P. aeruginosa* is a common cause of nosocomial infections and ventilator-associated pneumonia characterized by high fatality rates.^11^ *P. aeruginosa* pneumonia usually occurs in mechanically ventilated or immune-compromised patients and shows the classic hemorrhagic and necrotizing lung pathology.^12^^,^^13^

*P. aeruginosa* is associated with a higher mortality rate than other pneumonia pathogens and has many factors contributing to its pathogenesis and ability to colonize the host. It can form biofilms around bacterial colonies that can coat mucosal surfaces or invasive devices and is a factor for bacterial persistence.^11^ In addition, secreted lipoxygenase enzymes interfere with the host lipid signaling and modulate bacterial invasion.^14^ It also produces bioactive lipid mediators that inhibit the expression of major chemokines and the recruitment of key leukocytes.^14^ The determinants attributed to virulence of *P. aeruginosa* are the adhesins, pili, polysaccharide capsules, biofilm, production of toxins, elastase, protease, and hemolysins. In addition, it has intrinsic and acquired antibiotic resistance mechanisms such as plasmid-mediated and adaptive resistance.^15^ Other factors are quorum sensing, the transition from motility and stringent response, and persistence.^15^ It can perceive and process environmental changes in order to orchestrate physiological changes that promote adaptation to unfavorable conditions.^15^

As the bacteria can colonize hospitalized patients, especially those ventilated, surface and tracheal aspirate cultures’ reliability is poor in determining active infection. Therapy for *P. aeruginosa* pneumonia is often challenging, especially in strains of MDR. *P. aeruginosa* can carry genes encoding carbapenemases, and hence therapy for *PA* is often challenging due to the frequency of MDR.^4^^,^^5^^,^^13^^,^^16^^,^^17^ New Delhi metallo-beta-lactamase, Verona integron-encoded metallo-beta-lactamase, carbapenemase and imipenemase are the common carbapenemases encountered.^7^

In the patient described above, extreme prematurity and mechanical ventilation are the likely associated factors of the compromised host. Blood cultures and lung culture at autopsy grew *P. aeruginosa,* in addition to the blood culture collected prior to the patient’s demise. Sensitivity testing did not show MDR and was sensitive to the common anti-pseudomonal agents with low MIC. Microscopic examination of the lungs shows severe necrotizing pneumonia, and no other focus of infection was found. This indicates that the primary infection was pneumonia with resultant bacteremia and sepsis.

## CONCLUSION

This case of fatal necrotizing pneumonia due to *Pseudomonas aeruginosa* in an extremely low-birth-weight infant illustrates the vulnerability of such infants and the virulence of *Pseudomonas aeruginosa*. Health care facilities need to create task forces for antibiotic stewardship and efforts undertaken to reduce the emergence of MDR.
